# Large Animal Models in Regenerative Medicine and Tissue Engineering: To Do or Not to Do

**DOI:** 10.3389/fbioe.2020.00972

**Published:** 2020-08-13

**Authors:** Iris Ribitsch, Pedro M. Baptista, Anna Lange-Consiglio, Luca Melotti, Marco Patruno, Florien Jenner, Eva Schnabl-Feichter, Luke C. Dutton, David J. Connolly, Frank G. van Steenbeek, Jayesh Dudhia, Louis C. Penning

**Affiliations:** ^1^Veterm, Department for Companion Animals and Horses, University Equine Hospital, University of Veterinary Medicine Vienna, Vienna, Austria; ^2^Laboratory of Organ Bioengineering and Regenerative Medicine, Health Research Institute of Aragon (IIS Aragon), Zaragoza, Spain; ^3^Department of Veterinary Medicine, Università degli Studi di Milano, Milan, Italy; ^4^Department of Comparative Biomedicine and Food Science, University of Padua, Padua, Italy; ^5^Clinical Unit of Small Animal Surgery, Department for Companion Animals and Horses, University of Veterinary Medicine Vienna, Vienna, Austria; ^6^Department of Clinical Sciences and Services, Royal Veterinary College, Hertfordshire, United Kingdom; ^7^Department of Clinical Sciences of Companion Animals, Faculty of Veterinary Medicine, Utrecht University, Utrecht, Netherlands

**Keywords:** large animal models, sheep, pig, horse, dog, regenerative medicine, tissue engineering, naturally occurring disease

## Abstract

Rapid developments in Regenerative Medicine and Tissue Engineering has witnessed an increasing drive toward clinical translation of breakthrough technologies. However, the progression of promising preclinical data to achieve successful clinical market authorisation remains a bottleneck. One hurdle for progress to the clinic is the transition from small animal research to advanced preclinical studies in large animals to test safety and efficacy of products. Notwithstanding this, to draw meaningful and reliable conclusions from animal experiments it is critical that the species and disease model of choice is relevant to answer the research question as well as the clinical problem. Selecting the most appropriate animal model requires in-depth knowledge of specific species and breeds to ascertain the adequacy of the model and outcome measures that closely mirror the clinical situation. Traditional reductionist approaches in animal experiments, which often do not sufficiently reflect the studied disease, are still the norm and can result in a disconnect in outcomes observed between animal studies and clinical trials. To address these concerns a reconsideration in approach will be required. This should include a stepwise approach using *in vitro and ex vivo* experiments as well as *in silico* modeling to minimize the need for *in vivo* studies for screening and early development studies, followed by large animal models which more closely resemble human disease. Naturally occurring, or spontaneous diseases in large animals remain a largely untapped resource, and given the similarities in pathophysiology to humans they not only allow for studying new treatment strategies but also disease etiology and prevention. Naturally occurring disease models, particularly for longer lived large animal species, allow for studying disorders at an age when the disease is most prevalent. As these diseases are usually also a concern in the chosen veterinary species they would be beneficiaries of newly developed therapies. Improved awareness of the progress in animal models is mutually beneficial for animals, researchers, human and veterinary patients. In this overview we describe advantages and disadvantages of various animal models including domesticated and companion animals used in regenerative medicine and tissue engineering to provide an informed choice of disease-relevant animal models.

## Introduction

The use of sentient animals for research purposes is a controversial topic, which has raised public and ethical concerns and is criticized by opponents claiming that animal models often do not generate appropriate benefit with regards to their potential risks and harm and as a consequence, are often ethically not permissible. The increasing status of pets as family members and corresponding high level of veterinary care for privately owned pets further amplifies the controversy over the use of animals for research purposes.

However, animal models are still an important and, at a regulatory level, a compulsory component of translational research, which cannot yet be replaced by *in vitro* experiments. Although *in vitro* models allow for systematic, standardized analysis of various cellular, biophysical and biochemical cues in a controlled environment, without the natural variability inherent to *in vivo* animal models, they can only offer an abstract insight into the pathophysiology of diseases and disorders. Therefore, while animal models cannot yet be replaced, the number of animals used should be reduced to a minimum and experiments involving animals should be optimized with regard to their translatability and the welfare of the animals.

However, to date a reductionist approach often using immature laboratory species is commonly employed (Jackson et al., 2017). Small rodent animals, specifically mouse and rat, are valuable for research into mechanisms of disease and fundamental biology, but findings from such small animal models often do not translate into human clinical applications (Prabhakar, 2012; Lorbach et al., 2015). Shanks et al. impressively illustrated the translational challenges, showing the difference in bioavailability of pharmaceuticals between humans, primates, dogs and rodents (Shanks et al., 2009). However, although awareness is increasing there is still a massive disproportion between rodent studies and large animal studies.

Therefore, the European Medicines Agency (EMA), the USA Federal Food and Drug Administration (FDA) and the International Society for Stem Cell Research (ISSCR) recommend the use of large animal models to evaluate efficacy, durability, dose response, degradation and safety of advanced therapeutic medicinal products (ATMPs)^1^ ^,2^. For successful and timely translation from animal models to regulatory approval and clinical application, a step-wise development using laboratory animals for screening and early development work, followed by a large animal model such as the pig, sheep or horse which offers a more realistic approach for late development and pivotal studies would be more appropriate (Hurtig et al., 2011).

Moreover, animals develop many naturally occurring (or spontaneous) diseases that are equivalent to human disease leading to the development of the “One Health One Medicine” concept which presumes that diseases in men and animals (mostly mammals) have similar aetiologies and pathophysiologies and require analogous therapeutic approaches. Hence, human and veterinary medicine can mutually benefit from research that applies a one health approach. Using large animal models with naturally occurring disease with a similar pathophysiology as in humans, allows study of not only new treatment strategies but also disease development and prevention at a relevant age. However, although using naturally occurring disease models best reflect disease complexity, standardization of disease grade and availability of sufficient clinical case numbers for recruitment into studies can be challenging.

In order to achieve the best output while following the three R’s principle (to **r**educe, **r**efine and **r**eplace animal models) of using the smallest possible number of animals, animal models need to be optimized to the greatest possible extent (Madden et al., 2012). They require careful selection and design to ensure they are fit-for-purpose and address both optimal predictive validity, as well as ethical, animal-welfare and societal considerations. Species, anatomic, physiologic, biomechanical aspects and their clinical relevance need to be considered.

Furthermore, knowledge regarding the epidemiology and natural history of diseases in different animal species, disease similarities to humans, availability of diagnostics, treatment options, and outcome measures as well as criteria defining species specific quality of life and functional parameters is important but still scarce in the scientific community. Other important considerations in using large animal models include availability, handling and economic concerns.

To optimize scientific output and translational potential with animal welfare needs, tight cooperation between basic science, human and veterinary medicine is necessary. The veterinary academic environment offers unique expertise to make that goal attainable to the highest standards. This includes the veterinary knowledge required to make a rational decision for the choice of animal model rather than being based on in-house availability.

There are several research groups which have a track record of developing preclinical large animal models, some of which have managed to translate their research into clinical applications (Kang et al., 2010, 2013; Mcilwraith et al., 2011, 2012; Godwin et al., 2012; Smith et al., 2013; Bach et al., 2017; Whitehouse et al., 2017; Goldberg et al., 2018; Tellegen et al., 2018; Broeckx et al., 2019; Tellegen et al., 2019). This − by no means exhaustive − list clearly demonstrates the collective efforts of the veterinary community to provide large animal models to be used in translational projects.

However, yet it is still often argued, that translational studies using large animal models are rare because they are complex, time-consuming, technically demanding, slow, and usually not suitable for mechanistic investigations. Nevertheless, because large animals better reflect the human body conformation and pathophysiology of certain naturally occurring diseases than rodent models, these studies are essential justifying the challenges and costs. Unfortunately, the added value of the clinical relevance of large animal models is often not appreciated by reviewers of manuscripts and grant applications are assigned low scores on the basis of lack of mechanistic insights and insufficient conceptual novelty. However, for a successful translation of tissue engineering and regenerative medicine research into clinical therapies, it is critical that this misperception is corrected.

It is the authors’ hope that this review, which introduces different large animal models, their naturally occurring diseases and their specificities, may stimulate biomedical researchers to look for the very best model possible for their specific research question and that it will encourage interdisciplinary cooperation to optimize the choice of disease-relevant animal models in the future. Deciding which animal model should be used in a particular study is first and foremost dependent on defining the specific question that needs to be answered. Only then can the pertinent benefits and drawbacks of individual models be considered and a decision made.

In this review, we focus on horses, sheep, dogs, cats and pigs as the most frequently used large animal models in research and do not include primates due to the ethical dimension and limited indications, which require their specific use. Using animals which are so similar to humans, raises serious ethical concerns. Therefore, the use of non-human primates is closely monitored and strictly regulated and much has been done to specifically safeguard these animals. The use of great apes has been completely prohibited. As long as non-human primates are used for medical research, the European Commission strongly advocates the “3Rs principle,” now a legal obligation embedded in the EU legislation to: Replace non-human primates with viable alternatives whenever feasible, Reduce the use of non-human primates and Refine scientific procedures and the care and treatment of the animals. Even phasing-out the use of non-human primates in Europe is discussed^3^.

## Why the Choice of Animal Models Is Crucial

**FIGURE 1:**
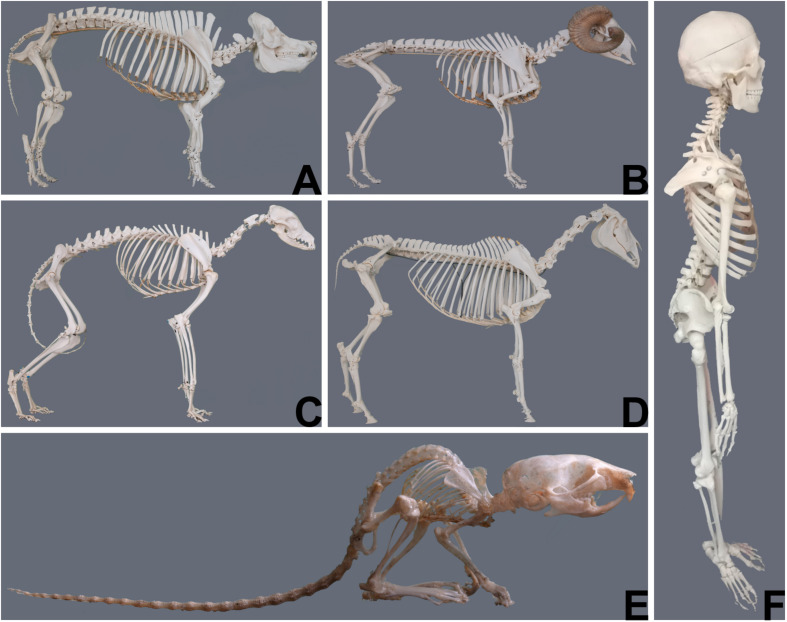
Gross anatomical differences between animals of different species (courtesy of Niklas Dresen, Institute of veterinary anatomy, University Leipzig) and the human (courtesy of Elfriede Cremer, Bernhard Cremer and Elisabeth Schieder). **(A)** Pig; **(B)** Sheep; **(C)** Dog; **(D)** Horse; **(E)** Mouse; **(F)** Human.

The most obvious and demonstrative reason why the choice of animal models is crucial, are gross anatomic differences between the human and different animals and even between animals of different species (Figure 1). These differences imply that the same anatomic structures may have a different function and are subjected to different biomechanical strains. Table 1 illustrates differences of different animals to emphasize the importance of correct model selection with respect to species’ physiologic aspects.

**TABLE 1:** Comparison of physiologic and biomechanical parameters of different model animals.

**Species**	**Mouse (C57BL/6)**	**Rat**	**Rabbit**	**Canine**	**Mini-Pig**	**Sheep**	**Goat**	**Horse**	**Human**
Body temperature (in °Celsius)	36.5−37.3°C	37.5−39.5	38.0−39.5	38.0−39	38.3−38.8	38.5−39.5	38.3−39.0	37.5−38.0	36.5−37.3
Heartrate (beats per minute)	491−626	250−450	150−300	60−160	68−72	60−80	60−80	28−40	60−80
Respiration rate per minute	272	70−120	35−100	15−30	14−18	16−30	10−30	10−14	12−20
Cartilage thickness medial femoral condyle (in mm)	0.1		0.3	0,6−1.3	1.5	0.7−1.7	0.7−1.5	1.75	2.35
Critical size cartilage defect (in mm)		1.4	3	4	6	7	7	9	10
Subchondral bone plate thickness medial femoral condyle (in mm)			0.4−0.5			0.7	0.3	0.7	0.2−0.5
Anterior cruciate ligament length (in mm)			10.6	21.2	37.0	31.77			37.78
Anterior cruciate ligament diameter (in mm)			4.84	5.45	10.86	9.27			12.73
Posterior cruciate ligament length (in mm)			10.0	22.8	39.68	37.03			40.30
Posterior cruciate ligament diameter (in mm)			4.34	5.33	8.2	6.67			14.23
Medial Meniscus length (in mm)			9.2	16.83	25.32	25.63			39.8
Lateral meniscus length (in mm)			10.0	16.3	25.60	26.03			33.28
Range of motion – knee joint extension (in °)			22	34	42	40	45		2.5
Range of motion – knee joint extension (in °)			161	160	144	146.7	145.5		137.5
Age of skeletal maturity (age until growths plates remain open)	3−6 months (growths plates remain open life−long)		16−39 weeks	12−24 weeks	42−52 weeks	36−48 months	36−48 months	60−72 months	18−22 years
Weight at skeletal maturity in kg	20−40 g	0.25−0.55	3−4	1−30	20−40	40−70	40−70	450−500	60−90
Life span in years	2	2.5−3.5		10−15	15	10−12	15−18	30	70−80

*Table modified from Tankersley et al. (1994); Campen et al. (2005), Frisbie et al. (2006); Baumgartner et al. (2009), Chu et al. (2010); Proffen et al. (2012), Maher et al. (2015); Moran et al. (2016) and https://www.jax.org/news-and-insights/jax-blog/2017/november/when-are-mice-considered-old# and https://www.jax.org/jax-mice-and-services/strain-data-sheet-pages/body-weight-chart-000664.*

### Age Matters

Age should be an important consideration in the choice of any animal model independent of the species used (Jackson et al., 2017). For practical and organizational reasons animal trials are often carried out in juvenile or neonatal animals. However, differences in the healing potential and therefore the healing response between juvenile and adult animals can bias the outcome of such trials (Namba et al., 1998; Beredjiklian et al., 2003; Conboy et al., 2005; Favata et al., 2006; Bos et al., 2008; Ansorge et al., 2012; Connizzo et al., 2013; Mienaltowski et al., 2016; Van Weeren and Back, 2016; Jackson et al., 2017). The use of skeletally mature animals of an appropriate age (Table 1) to mimic adult disease and healing potential is therefore a critical consideration for optimal study design. To truly reflect human age-related disease, the animals used should be of comparable age. Ideally old animals would be used to study age-related diseases as for instance senile osteoporosis. However, the use of elderly compared to immature or young adult animals requires specific considerations, as aged animals are more difficult to procure and may suffer from comorbidities. Hence, potential animal loss due to other diseases needs to be accounted for in the study design and financial planning. Furthermore, the predisposition for age-related diseases varies between species.

### Naturally Occurring and Generated Models of Genetic Disease

Many naturally occurring genetic diseases have been identified in companion and farm animals which are often caused by a mutation in an orthologous gene and lead to a comparable clinical phenotype as observed in human patients, including the pathological alterations at the biochemical and cellular levels (Lairmore and Khanna, 2014; Kol et al., 2015). Most of these animal models are associated with congenital heart disease, lysosomal storage disease, hemophilia, muscular dystrophies, neurological disorders, immunodeficiencies and dwarfism (Lairmore and Khanna, 2014). Information about these naturally occurring genetic diseases in animals was compiled in a comprehensive database − Online Mendelian Inheritance in Animal − created by Prof. Frank Nicholas at The University of Sydney and Australian National Genomic Information Service^4^.

### Naturally Occurring and Generated Models of Musculoskeletal Disease

Osteoarthritis (OA) is a heterogeneous disease for which no single animal model perfectly recapitulates the complex etiology and clinical manifestations of the human disease (Aigner et al., 2010; Cohen-Solal et al., 2012; Little and Zaki, 2012; Mccoy, 2015). Currently available OA models are generally grouped into spontaneous, or surgically induced models. Spontaneous models include naturally occurring disease or genetically manipulated models, whereas surgically induced models employ (i) destabilization of the joint such as partial or total meniscectomy, meniscal tear, anterior cruciate ligament or posterior cruciate ligament transection, medial and/or lateral collateral ligament transection or osteotomy (ii) physical defects of the articular cartilage such as creation of articular grooves, (iii) impact trauma including transarticular impact, and intra-articular osteochondral fragmentation (iv) chemically induced lesions using intra-articular injection of monosodium iodoacetate, collagenase, carrageenan or Freund adjuvant (Bentley, 1975; Little and Zaki, 2012; Lampropoulou-Adamidou et al., 2014; Mccoy, 2015). Spontaneous models that develop progressive and chronic disease are likely to more closely mimic idiopathic OA. However, these models take longer to develop and tend to be more variable with respect to outcome measures (Vincent et al., 2012; Teeple et al., 2013; Lampropoulou-Adamidou et al., 2014; Mccoy, 2015).

Surgical models have the advantage of repeatability and reproducibility as well as rapid onset and progression (Lampropoulou-Adamidou et al., 2014), but for that reason are less ideal models of spontaneous OA and are often regarded as posttraumatic (secondary) OA (Bendele, 2001; Little and Hunter, 2013; Teeple et al., 2013; Mccoy, 2015).

The validity of chemically induced models for OA has been questioned (Poole et al., 2010; Teeple et al., 2013) due to the resulting widespread cell death and rapid joint destruction, which are not considered typical for either spontaneous or posttraumatic OA (Little and Zaki, 2012).

Animal models are further widely used in osteoporosis research. They include, among others, models for disuse induced osteoporosis, glucocorticoid-induced osteoporosis and postmenopausal osteoporosis. The most popular animal models of postmenopausal osteoporosis are those generated in the mouse, rat, sheep, and nonhuman primates by ovariectomy (Iwaniec, 2008).

The choice of the animal models differ markedly, depending on the objectives of the study. It has to be noted that rodents for example are of limited value for investigating intra−cortical bone remodeling, because they lack true Haversian cortical bone remodeling under physiological conditions due to their small weight (Baron et al., 1984; Lelovas et al., 2008; Iwaniec, 2008). Larger animals such as dogs are more appropriate for these studies because, similar to humans, dogs have well-developed Haversian remodeling (Iwaniec, 2008).

### Challenges of Translating Results From Animal Models to Human Patients – An Example

To date, animal models of human asthma have included: Drosophila, rats, guinea pigs, cats, dogs, pigs, cattle, sheep, horses and primates, but the most widely used model is the mouse (Zosky and Sly, 2007; Kirschvink and Reinhold, 2008; Shapiro, 2008; Blume and Davies, 2013). The mouse is a useful model due to the availability of specific probes and reagents for studying allergic outcomes, such as cellular and humoral responses, and the good adaptability for genetic manipulation (Shapiro, 2008; Bonamichi-Santos et al., 2015). Nevertheless, this model has some limitations for translational medicine mainly related to the anatomical and physiological differences with respect to man. Obviously, the lung and bronchial tree, total lung capacity (6 liter for man vs. 1 ml for mouse), and the blood-gas barrier thickness (0.62 μm vs. 0.32 μm) are much smaller than in man and the bronchial artery supplies the entire lung in man but is absent in the pleura, septa and alveoli in mice. In addition, the respiratory rate, or beats per minute (10−14 vs. 250−350), is very different in people and mice. Moreover, the mouse lacks sub-mucosal glands and has limited airway smooth muscles compared to man (Lange-Consiglio et al., 2019). In view of these important differences, the pre-clinical results obtained when using a mouse model for asthma should be interpreted with care. Furthermore, the mouse does not have natural inflammatory or allergic pulmonary pathologies, so airway inflammation is usually induced by exposure to ovalbumin (OVA) or other aeroallergens. In contrast to naturally occurring human asthma, which is a chronic disease characterized by persistent inflammation and remodeling due to intermittent or continuous inhalation exposure to allergens resulting in chronic eosinophilic/neutrophilic inflammation (Aun et al., 2017; Bullone and Lavoie, 2019), the mouse model shows more acute (<3 months) inflammation and no remodeling. To circumvent this problem, systemic sensitization protocols and repeated exposures to allergens have been tried but the results obtained from different routes of systemic sensitization (subcutaneous injection, intraperitoneal injection or intranasal inhalation) and different allergens (OVA, fungi, *Ascaris* antigens, house dust mite, cockroach extracts), used alone or in combination, are difficult to compare and to interpret (Aun et al., 2017). For example, in the mouse the induced inflammation profile, although dependent on the antigen, is mainly Th2, mirroring disease in only a subsection of human asthmatics who are Th2 and/or Th1/Th17 (Douwes et al., 2002; Woodruff et al., 2009). Another criticism of the mouse model is that OVA does not induce asthma in human patients and the sensitization routes do not mimic the routes of exposure to allergens in human asthma (Aun et al., 2017). Hence, differences in the results may be due to the different types of allergens and sensitization routes.

An appropriate animal model for translational studies should mimic the pathological changes associated with human asthma and reflect the environmental factors that determine the evolution of human asthma.

## Horses as Animal Models

### General Considerations

Horses (*equus caballus*) are a well-accepted, well-established and clinically relevant animal model particularly for musculoskeletal disease, which is of major interest in regenerative medicine.

An important aspect of clinical research is the precise demonstration of the initial injury, the disease progress, outcome and follow up. The validated applicability of advanced diagnostic methodologies in horses such as arthroscopy and MRI (together with scoring approaches) (Brittberg and Winalski, 2003; Marlovits et al., 2006), ultrasound, radiographs, CT and scintigraphy, has made the horse a popular model for which non-terminal studies with thorough evaluation and monitoring are possible. Also, second-look arthroscopy and serial sampling are feasible. Moreover, the large size of horses allows for the creation of critical size defects or multiple defects and offers a high amount of material that can be sampled for analysis. This enables large and comprehensive studies which may not be possible in smaller animals. Together with well-established histologic scoring (Mcilwraith et al., 2010) and pain scores (Price et al., 2003; Graubner et al., 2011; Dalla Costa et al., 2014; Gleerup et al., 2015) or assessment of other clinical parameters for horses these methods facilitate comparability of diagnosis, follow-up and results. Controlled postoperative exercise programs and rehabilitation protocols using e.g., treadmills and horse walkers further support standardization of the results. A broad offer of modern methods to further objectify outcome measures became available including gait kinematics (e.g., lameness locators) and/or kinetics using force plate/ground reaction force analysis.

Also the lack of traceability of cells injected for cell therapies could be overcome to a certain extent by using either super paramagnetic iron oxide particle (SPIO) for MRI (Delling et al., 2015a,b; Julke et al., 2015; Berner et al., 2016; Burk et al., 2016) or nuclear labeled (Technetium 99M, GFP, Indium 111) cells for scintigraphic tracing (Sole et al., 2012, 2013; Becerra et al., 2013; Trela et al., 2014; Dudhia et al., 2015; Spriet et al., 2015; Espinosa et al., 2016; Geburek et al., 2016; Scharf et al., 2016).

Some disadvantages of using the horse as a model include high costs of animal of animal purchase, maintenance/handling as well as ethical concerns and lower acceptance of the horse as an experimental animal compared to small animal studies by the lay public. In addition, some key parameters building the framework used in studies applying Omics approaches are not well enough researched in horses yet. A restrictive annotation status and availability of equine specific antibodies, molecular tools and markers are limiting factors. A major challenge when using horses is that their weight precludes non-weight-bearing investigations postoperatively. Significant limitations may arise regarding biomechanical strains, which far exceed those considered physiologic in humans and other animal models, which could render the stabilization of injured structures, transplants and/or sutures ineffective. Therefore, horses are a less amenable model for meniscus or bone repair. Nonetheless, these are major challenges in equine patients and several different attempts have been made or are envisaged to support healing of these structures by regenerative medicine approaches (Fox et al., 2010; Milner et al., 2011; Ferris et al., 2012; Kisiday et al., 2012; Mcduffee et al., 2012; Seo et al., 2014; Warnock et al., 2014; Govoni, 2015; Yu et al., 2015; Gonzalez-Fernandez et al., 2016) which may also hold valuable preclinical results for human medicine. For example, hyperextension of the stifle joint was found to lead to pathologic levels of forces and injury in the cranial horn of the equine medial meniscus, analogous to observations in the human posterior medial horn upon hyperflexion (Drosos and Pozo, 2004).

### Tendinopathy

Horses commonly suffer from naturally occurring tendon injuries (tendinopathy) and degenerative joint disease (osteoarthritis − OA) with similar pathophysiology to the human in terms of etiology and risk factors, which include over-exercise, age and genetic factors (Goodship et al., 1994; Patterson-Kane and Firth, 2009; Mcilwraith et al., 2012; Voleti et al., 2012; Smith et al., 2014; Andarawis-Puri et al., 2015). As athletic individuals, horses incur idiopathic primary or sports related injuries to tendon and joint related tissue.

An example is the equine superficial digital flexor tendon (SDFT) which performs a similar function to the human Achilles tendon during high-speed locomotion. In both species, their respective tendons are one of the most frequently injured (Jarvinen et al., 2005; Thorpe et al., 2010) with age and participation in sports as key risk factors. The SDFT supports the metacarpophalangeal (MCP) joint and functions as an energy storing elastic tissue to enable efficient locomotion. During high-speed locomotion the SDFT can experience strains of 16% as the MCP joint hyperextends. These strains are within the functional limit of the SDFT at which failure can occur (Richardson et al., 2007). The Achilles tendon can experience strains of up to 8% allowing as much as 34% of the total work performed by the calf muscles to be stored in the Achilles (Fukashiro et al., 1995).

Acute and chronic Achilles tendon pathology is estimated to be responsible for as many as 50% of all sports-related injuries in humans (Fukashiro et al., 1995; Maffulli, 1999; Jarvinen et al., 2005). The incidence of SDFT tendinitis in horses is reported to be as high as 8−43% (Dowling et al., 2000). Injuries in both often manifest within the body of the tendon as core lesions, which heal by the formation of fibrous scar tissue. This scar tissue is biomechanically inferior with significantly reduced elasticity which leads to a high risk of re-injury (Smith, 2008). It is therefore essential that repair strategies are aimed at restoring function by achieving scar-free healing for which regenerative medicine holds great potential. Studies in the horse to test and improve cell and cell free therapies for tendon regeneration (Smith et al., 2003; Pacini et al., 2007; Richardson et al., 2007; Schnabel et al., 2007; Fortier and Smith, 2008; Lacitignola et al., 2008; Smith, 2008; Godwin et al., 2012; Marfe et al., 2012; Carvalho Ade et al., 2013; Renzi et al., 2013; Smith et al., 2013; Van Loon et al., 2014; Geburek et al., 2015; Muttini et al., 2015) could serve as preclinical data for human medicine.

However, due to the challenges of standardization of disease grade and availability of sufficient clinical case numbers for recruitment of horses with naturally occurring disease, a number of induced equine models have been developed to investigate both tendon and joint disease.

Several surgically induced tendon injury models have been developed to try to achieve a standard lesion size, anatomical location and the ensuing inflammatory response as well as time to treatment (Guest et al., 2008; Schramme et al., 2010; Caniglia et al., 2012; Cadby et al., 2013). While most of these are aimed at partial or full transection of the tendon, the mechanically induced model described by Schramme et al. (2010) mimics a typical tendon core lesion of spontaneous disease with similarities in healing characteristics (Cadby et al., 2013). In contrast, collagenase induced tendon injury models which attempt to mimic core lesions (Williams et al., 1984; Nixon et al., 2008; Moraes et al., 2009; Schnabel et al., 2009; Crovace et al., 2010; Karlin et al., 2011; Watts et al., 2011, 2012; Carvalho Ade et al., 2013) lead to a strong inflammatory response and are difficult to standardize with respect to location, size, shape and volume due to leakage of collagenase through the injection sites and uncontrollable diffusion from the center of the tendon (Schramme et al., 2010).

### Cartilage Injuries and Osteoarthritis

Another example to illustrate “what the horse can tell the human” is Osteoarthritis, a degenerative joint disease characterized by progressive loss of articular cartilage. Adult articular cartilage has limited capacity for repair and regeneration (Kim et al., 1991). Any disruption of the superficial zone, or injury to the chondrocytes that maintain the cartilage matrix and zonal architecture, affects the load-distribution of the viscoelastic hyaline cartilage and may ultimately culminate in degenerative joint disease (Rolauffs et al., 2010). OA of the knee and hip joints is one of the most commonly diagnosed diseases in human general practice with 52 million people (=22.7% of adults older than 18 years) in the United States and an estimated 30 – 40 million Europeans suffering from arthritis of one or more joints (Cheng et al., 2012; Johnson and Hunter, 2014). With age and obesity as key risk factors the prevalence of OA is expected to double by the year 2020 (Johnson and Hunter, 2014). As currently no proven disease-modifying therapy capable of restoring damaged articular cartilage and function of the joint is available, there is an increasing demand for novel, safe and effective treatments, which regenerative medical research could offer. In equids as for human patients, there is an unmet need for early diagnosis and effective treatments that allow return to full performance (Mccoy, 2015). In horses OA constitutes the main cause of chronic lameness with an incidence of chronic degenerative joint disease in elderly horses of up to 83.5%. Interestingly, not only is the pathophysiology of equine OA similar to the human but also the thickness of the knee cartilage is similar to the human (Frisbie et al., 2006; Malda et al., 2012). These similarities support the horse as relevant model for studies on naturally occurring OA.

A number of surgically induced equine models of articular cartilage degeneration and healing have been developed which were reviewed by Mcilwraith et al. (2011). As in humans, the major aims of OA research are to achieve resurfacing of the damaged cartilage with biomechanically resilience and acceptable pain control. However, for any studies on cartilage repair it is important that the duration should be at least 8 to 12 months, as failure at long-term follow-up is a common outcome in human and equine clinical trials even if short-term results look promising in animal models (Mcilwraith et al., 2011).

### Asthma

Horses, analogous to humans, commonly suffer from asthma. Asthma is a chronic inflammatory disease characterized by airway hyper-responsiveness and airway remodeling due to increased mucus production, epithelial fibrosis, hypertrophy and hyperplasia of airways smooth muscles, and gland enlargement (Shinagawa and Kojima, 2003). This remodeling can induce irreversible obstruction of airways and may be a consequence of chronic tissue inflammation and altered repair processes. Since function and structure are closely related, the hypothesis is that remodeling leads to loss of airway and lung function (Bullone and Lavoie, 2019).

Around 300 million people worldwide (both adults and children) suffer from asthma and hence the societal impact is high^5^. The standard therapy is based on corticosteroid administration to reduce airway obstruction thus improving quality of life. However, about 20% of people are corticosteroid resistant and do not respond to therapy (Panettieri, 2016). Corticosteroid therapy is reparative and not regenerative and does not counteract remodeling. Better therapies may be derived from a regenerative approach to asthma-induced pathology.

The gold standard species for studies into human asthma would be human patients, but such studies are ethically impossible, because of the large number of patients requiring repeated biopsies to understand the causes of remodeling. Therefore, although requiring ethical authorizations, animal models are essential to advance understanding of the disease.

Severe equine asthma (SEA), which occurs spontaneously in horses (Herszberg et al., 2006; Williams and Roman, 2016), shares many features with human asthma. The horse has potential to be a good animal model with similar lung anatomy to man. SEA shares many features with human asthma: lower airway inflammation, completely reversible airflow obstruction, bronchial hyperresponsiveness, increased respiratory efforts at rest, coughing and exercise intolerance (Bullone and Lavoie, 2015; Couetil et al., 2007). This condition is spontaneously triggered by exposure to environmental antigens present in horse housing, similar to exposure in man and it can become incurable like chronic asthma in people. Up to 10–15% of adult horses suffer from SEA (Hotchkiss et al., 2007) with a Th-2 predominant cytokine profile (increase of IL-4), as described in human asthma (Lavoie et al., 2001; Klukowska-Rotzler et al., 2012), and decrease of Th-1 profile (decrease of interferon-γ). The predominant cell type in bronchoalveolar lavage fluid (BALF) found in horses may be different to humans depending on the severity of asthma: horses with severe and late-onset asthma have neutrophilic inflammation (Panettieri, 2016) as demonstrated in some people (Cosmi et al., 2016), while increased eosinophils are frequently detected in milder forms of equine asthma (Couetil et al., 2007). As in people with neutrophilic asthma, horses with SEA can show an increase in Th-17 expression (Debrue et al., 2005; Cosmi et al., 2016).

In a good animal model, homology of genes regulating immune function is essential and the horse shares higher homology with man for *IL2, IL23*, and *IL17*, compared to the mouse (Tompkins et al., 2010; Lange-Consiglio et al., 2019). However, the most interesting aspect of the horse as a model to study asthma is airway remodeling, although this is less marked and involves the bronchial tree more peripherally than in man (Bullone and Lavoie, 2019). The remodeling can be completely reversed by appropriate corticosteroid treatment in both human patients and horses (Bullone and Lavoie, 2019) and sequential biopsies can be collected from the same standing sedated horse without the imperative to sacrifice the animal as compared to the mouse (Leclere et al., 2011).

### Additional Considerations Regarding Horses

In horses’ wounds on the distal limbs show delayed healing compared to wounds located on the upper body.

Reasons for this are not fully understood. However, differences in the rate of epithelization and wound contraction, inefficient inflammatory response (resulting in chronic inflammation and hence impaired formation of healthy granulation tissue), imbalance in collagen homeostasis, profibrotic environment, tissue hypoxia and inappropriate cell apoptosis are discussed as contributing factors (Provost, 2019).

Interestingly ponies heal better and faster than horses, with ponies yielding a quicker and more intense inflammatory response and an improved resistance to infection as compared to horses (Provost, 2019). Some of the most important advantages and disadvantages of using horses as model animals are summarized in Table 2.

**TABLE 2:** Advantages and disadvantages of the horse as a model.

**Advantages**	**Disadvantages**
Largest of the models: Multiple large (critical size) defects and serial sampling possible	Ethical concerns – companion animals
Functional correspondence of equine SDFT and human Achilles tendon	Different breeds, not usually purpose-bred for research
Thickness of the knee cartilage is similar to the human	Costs- Special facilities needed for housing, surgery, imaging, necropsy, etc.
Imaging plus validated scoring approaches	Non-weight-bearing postoperatively is not feasible
Arthroscopy (also second look) plus validated scoring approaches	Restrictive annotation status and availability of equine specific antibodies, molecular tools and markers
Controlled postoperative exercise programs and rehabilitation using treadmills and horse walkers to support standardization are well established	Weight − biomechanical strains, which far exceed those considered physiologic in humans
Well characterized temporal pattern of healing	
Equine aging well documented	
Clinical need − naturally occurring disease	

Another challenge of using horses as animal models, particularly for orthopedic disease, is so called supporting limb laminitis (SLL). Laminitis is a disorder of the tissue suspensory apparatus which suspends the distal phalanx to the inside of the horse’s hoof wall. SLL of the contralateral or supporting limb occurs when horses are forced to bear weight predominantly unilaterally (with the supporting limb) for prolonged periods, due to a severe, unilateral lameness. Mechanical loading or overloading of the supporting limb is the primary factor in its pathogenesis (Baxter and Morrison, 2008; Orsini, 2012).

## Sheep as Animal Models

### General Considerations

Domestic sheep (*Ovis aries*) provide unique opportunities in research as an experimental and pre-clinical animal model (Hems and Glasby, 1992; Glasby et al., 1993; Al Abri et al., 2014) because of their availability, low costs and acceptance by the society as a research animal (Diogo et al., 2017). Sheep are docile, easy to handle and relatively inexpensive with respect to housing and feeding. Their size (50−90 kgs) is more similar to humans than small animal models, lending themselves to repeated sampling from different anatomical structures over an extended period. Their size is ideal for clinical imaging modalities designed for humans such as MRI or CT (which are limited with other large animal models like the horse). At the same time, it allows for testing surgical procedures and medical devices in animals similar to human-size (e.g., bioengineered constructs, pacemakers, stents). On the other hand, sheep housing requires more space (barns for pens) which are not widely available. The commercial availability of molecular tools (e.g., antibodies) is also more limited than for rodents although these are increasing. Nonetheless, the practical disadvantages of the sheep as an experimental model do not make it inaccessible. Based on the aim of the study, the potential benefits may compensate its technical limitations. The publication and annotation of the sheep genome (Jiang et al., 2014) should improve the amount of commercially available reagents, thus facilitating the use of the ovine model in future studies. Concomitantly, the annotation of the sheep genome could support the development of useful biological tools for sheep as genetic models of human diseases (e.g., Huntington’s Disease) (Pinnapureddy et al., 2015). Moreover, anesthesia and surgical equipment in sheep is more similar to humans than other large animals (like horses) and small rodents: Hence, using sheep does not require significant investment in large and specialized handling equipment, or surgical tables. At the same time, sheep can be sourced relatively easily and at low cost and they are considered as a socially acceptable animal model for research that raises fewer ethical issues than companion animals (Entrican et al., 2015; Rogers, 2016).

Sheep are used as models for a wide range of pathologies: cardiovascular diseases (Divincenti et al., 2014; Rabbani et al., 2017), orthopedics (Kon et al., 2000; Vandeweerd et al., 2013; Dias et al., 2018; Mcgovern et al., 2018; Music et al., 2018), respiratory function (Meeusen et al., 2009) and reproductive or pregnancy disorders (Andersen et al., 2018; Morrison et al., 2018). A major reason is that ruminants, as compared to rodents, share more anatomical and physiological characteristics (with exception of the digestive tract − testing efficacy of drugs may be complicated by the 4-stomach system and uptake dynamics which defer from human gastrointestinal tract characteristics) with humans (Scheerlinck et al., 2008). This makes the sheep a useful model for preclinical and translational studies in fields of Tissue Engineering and Regenerative Medicine.

### Musculoskeletal Disorders

Sheep have anatomical and biomechanical features relatively similar to humans (bone composition, weight, joint structure and architecture) which allows for good simulation of healing and remodeling processes of bone or cartilage tissue (Newman et al., 1995; Taylor et al., 2006). In addition, arthroscopic evaluation is possible in the sheep due to the size of their stifle joints. Therefore, the ovine species is the most commonly used large animal model in orthopedic research including studies on: cartilage repair (Music et al., 2018), meniscal repair (Hurtig et al., 1998; Tytherleigh-Strong et al., 2005), osteochondral tissue engineering (Sanjurjo-Rodriguez et al., 2017), tendon defects (Crovace et al., 2008; Martinello et al., 2013), osteoarthritis (Oakley et al., 2004; Gugjoo et al., 2019), and osteoporosis (Dias et al., 2018) among the others.

Sheep have been involved in studies for treating critical-sized bone defects using scaffolds with or without Mesenchymal Stem cells (MSCs). These treatments were shown to enhance bone formation and improve mechanical properties if compared to gold standard reparative methodologies like bone grafts (Kon et al., 2000; Cipitria et al., 2013; Fernandes et al., 2014; Berner et al., 2015; Mcgovern et al., 2018; Pobloth et al., 2018).

Although the ovine knee cartilage differs in thickness to human cartilage (0.7−1.7 mm and 2.35 mm, respectively), it provides a close match regarding mechanical properties for preclinical studies (Frisbie et al., 2006; Chu et al., 2010; Mclure et al., 2012). Tissue engineering approaches including different cell sources (as MSCs or chondrocytes) have been widely tested in the sheep for chondral/osteochondral defects (Lo Monaco et al., 2018; Gugjoo et al., 2019). Cells can also be applied with scaffolds of different nature to improve and support regeneration (Chitosan, type I/III collagen, b-TCP, collagen hydrogels) (Bernstein et al., 2013; Sanz-Ramos et al., 2014; Dias et al., 2018). For example, Hopper et al. (2015) used a biphasic collagen-GAG scaffold loaded with MSCs in a full-thickness osteochondral defect boosting cartilage repair while Zorzi et al. (2015) used a 1:1 chitosan-collagen scaffold seeded with human MSCs for articular cartilage regeneration (Hopper et al., 2015; Zorzi et al., 2015). Recently, a bilayered scaffold to simulate the bone-cartilage interface (chondral and bone tissue components) has been developed and tested in sheep (Schagemann et al., 2009; Fan et al., 2013).

Furthermore, regenerative strategies for osteoarthritis (usually induced by meniscectomy) have been investigated in sheep (Song et al., 2014; Desando et al., 2016; Feng et al., 2018). Of particular interest are the studies on scaffolds for meniscal repair because of its shared characteristics with the human meniscus (cellularity, vascularity, biomechanics) (Chevrier et al., 2009; Brzezinski et al., 2017). Gruchenberg et al. (2015) tested a silk fibroin scaffold as a meniscal implant after meniscectomy in sheep showing its biocompatibility (Gruchenberg et al., 2015).

Spontaneous cartilage lesions (including osteoarthritis) have been observed in the sheep without experimental induction (Hurtig et al., 2011; Vandeweerd et al., 2013; Kuyinu et al., 2016). These are especially prevalent in aging sheep and might better recapitulate the human ailment than artificially created cartilage defects.

Sheep, like horses, are ideal candidates for tendinopathy modeling, but cheaper and easier to handle and house. Martinello et al. (2013) showed the treatment efficacy of MSCs, with or without PRP (platelet rich plasma), on collagenase-induced tendinitis in the superficial digital flexor tendon, with a better structural organization of the repaired tendon (Martinello et al., 2013). Deprés-Tremblay et al. (2018) tested the use of chitosan-PRP implants in an ovine acute defect model to mimic rotator cuff injuries. The implants led to an extensive bone remodeling and tissue ingrowth at the tendon-bone interface level (Deprés-Tremblay et al., 2018).

### Nervous System

The ovine species also serves as an adequate and effective model to study peripheral nerve regeneration, because of the similar nerve size (Starritt et al., 2011) and similar regenerative behavior (Hems et al., 1994; Fullarton et al., 2001) compared to humans (Diogo et al., 2017).

Apart from conventional autografts and allografts for repairing peripheral nerve injuries in sheep (Frey et al., 1990; Matsuyama et al., 2000), tissue engineering techniques have also been applied. Casanas et al. (2014) applied a commercially available biodegradable scaffold with MSCs or PRP to reconstruct damaged radial and tibial nerves. The addition of MSCs, with or without PRP, led to the production of myelinated nerve fibers at the distal and proximal level with fiber regeneration and functional recovery after 6 months (Casanas et al., 2014). Radtke et al. (2011) compared the use of autologous nerve and acellularized vein grafts produced from spider silk. The outcomes obtained with the construct where similar to the nerve autograft results: axonal regeneration and myelination were achieved at 10 months (Radtke et al., 2011).

Using sheep models, MSCs were shown to play a reparative role in intervertebral disc regeneration. Injection of MSCs led to a reduction of degeneration of the discs compared to the control group (Freeman et al., 2016; Daly et al., 2018).

### Heart Disease

Sheep have been frequently used as model for cardiovascular applications, especially for testing heart valves which have similar valve anatomy to the human and the sheep size permits access to the pulmonary and aortic valve. Kluin et al. (2017) developed an *in-situ* heart valve replacement for the pulmonary valve using a resorbable synthetic graft. 12 months post-implantation the tissue-engineered valve was shown to be colonized by host cells and replaced by newly formed tissue with a mature organization of the extracellular matrix without any sign of valve calcification (Kluin et al., 2017).

Cell therapies with MSCs have further been applied in acute myocardial infarction models to improve myocardial function. The inoculation of cells has been demonstrated to be safe, to increase vasculature, and to reduce fibrosis in the infarcted heart (Houtgraaf et al., 2013). Rabbani et al. (2017) showed that the injection of MSCs and endothelial cells (ECs) promoted angiogenesis and cardiac function, supposing that one of the mechanisms of action of the MSCs might lie in their differentiation potential toward the endothelial lineage (Rabbani et al., 2017).

Also, different tissue engineering approaches for the development of preclinical vascular grafts have been tested in the sheep model (Cummings et al., 2012; Aper et al., 2016; Fukunishi et al., 2016; Koobatian et al., 2016).

### Tissue Engineering Applications in Other Systems

The ovine model has further been deployed to test regenerative approaches for treating respiratory disorders (similar airways structure and lung size to humans):

MSCs led to a reduction of inflammation and oedema and an improved oxygenation in sheep models of acute respiratory distress (Asmussen et al., 2014; Kocyildirim et al., 2017). In an induced emphysema model, the infusion of MSCs resulted in blood reperfusion of the damaged tissue and the formation of new extracellular matrix (Ingenito et al., 2012).

Recently, Kajbafzadeh et al. (2019) have tested the transplantation viability of decellularized kidneys in sheep.

The sheep model has also been described for wound healing studies because it allows for the creation of relatively large and deep wounds to mimic the typical scenario of traumatic injuries like burn injuries or decubitus ulcers. Martinello et al. (2018) used a sheep second intention wound healing model and showed how the intradermal and topical application of allogeneic MSCs led to a better re-epithelialization and dermal structure as compared to the control group at 42 days after wounding (Martinello et al., 2018). The identical model was recently used by Iacopetti et al. (2020) to compare secondary intention healing of wounds, treated with a topical application of commercially available hyaluronic acid, Manuka honey or Acemannan gel (Iacopetti et al., 2020).

In a similar ovine wound model, Liebsch et al. (2018) applied native spider silk as a wound dressing to test its biocompatibility and regenerative capacities (Liebsch et al., 2018). Mazzone et al. (2020) used bioengineered autologous skin substitutes to treat myelomeningocele in a spina bifida repair model. The skin substitute, made of hydrogel colonized by autologous fibroblasts and keratinocytes, was transplanted in utero. The skin substitutes showed a normal histology after 1 month (Mazzone et al., 2020).

Recently, Martines et al. (2020) evaluated the use of a low-temperature atmospheric pressure plasma (ionized gas) as a treatment for extensive wounds in a sheep model. The plasma stimulated cell proliferation, angiogenesis and the development of skin adnexa; concomitantly, it reduced bacterial infection and inflammation (Martines et al., 2020).

A different tissue engineering approach to treat myelomeningocele was used by Watanabe et al. to treat spina bifida wounds with a gelatin/collagen sponge hybrid scaffold (Watanabe et al., 2016).

### Embryonic/Fetal Healing

True “scarless healing” is observed only in embryos and early fetus (Stramer et al., 2007). The restitutio ad integrum in embryos (Beredjiklian et al., 2003) is considered an ideal situation unmatched by any treatment regimen in adults. Therefore, an increasing amount of research studies is performed in embryos or fetal animals. To study the mechanism of fetal regeneration, relevant *in vivo* as well as *in vitro* models are required. Fetal sheep share many important physiological and developmental characteristics with humans and have hence proven themselves invaluable models for mammalian physiology (Almeida-Porada et al., 2004; Jeanblanc et al., 2014). Sheep frequently carry twins, which allows using one twin as uninjured control on a background of low genetic variation to enable differentiation between regular fetal development and fetal response injury.

Furthermore, their long gestational period (150 days) provides sufficient temporal resolution to translate findings obtained in sheep into human parameters (Almeida-Porada et al., 2004; Jeanblanc et al., 2014).

Fetal sheep have a fully functioning immune system by 75 days of gestation (gd) (Emmert et al., 2013). They produce leukocytes by 32 gd (Sawyer et al., 1978), TNF and Il-1 as early as 30−40 gd (Dziegielewska et al., 2000) and obtain the capability to form significant amounts of specific antibodies in response to antigenic stimulation as early as 70 gd (Silverstein et al., 1963). Fetal lambs reject orthotopic skin grafts and stem cell xenotransplants placed post 75−77 gd (Silverstein et al., 1964) and mount an inflammatory response to injury by gestational day 65 (Nitsos et al., 2006; Moss et al., 2008; Herdrich et al., 2010; Morris et al., 2014).

For all these reasons, results obtained in the fetal lamb have been directly applicable to the understanding of human fetal growth and development and are highly predictive of clinical outcome in a variety of applications including in utero stem cell transplantation (Liechty et al., 2000; Almeida-Porada et al., 2004, 2007; Porada et al., 2005; Kuypers et al., 2012; Kim et al., 2013; Jeanblanc et al., 2014).

### Additional Considerations Regarding Sheep

Due to their special stomach system (4 stomachs: rumen, reticulum, omasum, and abomasum) bio-availability and efficacy of drugs administered orally is questionable for the human GI tract. Moreover, prolonged inappetence and application of non-steroidal anti-inflammatory drugs, antibiotics or both resulting in sustained high acidity in the abomasum may cause abomasal ulceration. Also stress, high dietary fiber and inadequate dietary fiber are believed to play a role (Ducharme, 2004; Fubini and Ducharme(eds), 2004).

Therefore, pain management and anti-microbial management have to be planned carefully and adapted to meet the special requirements of sheep (Lizarraga and Chambers, 2012; Varcoe et al., 2019). Sheep guidelines for pain assessment by facial expression are available (Hager et al., 2017) which may help managing pain.

Some of the most important advantages and disadvantages of using sheep as model animals are summarized in Table 3.

**TABLE 3:** Advantages and disadvantages of the sheep as a model.

**Advantages**	**Disadvantages**
Multiple large (critical size) defects and serial sampling possible	Different breeds, not usually purpose-bred for research
Thickness of the knee cartilage is similar to the human	Special facilities needed for housing, surgery, imaging, necropsy, technical skills
Docile to handle	Non-weight-bearing postoperatively is not feasible but can modulate with location
Availability, and acceptance by the society as a research animal	Different stomach system than humans
Size is more similar to humans than small animal models or horses	Ethical concerns but minor compared to companion animals
Publication and annotation of the sheep genome	Costs
Imaging plus validated scoring approaches available (esp. for orthopedics)	
Arthroscopy (also second look) plus validated scoring approaches	

## Pigs as Animal Models

### General Considerations

Porcine models present the advantage of having similarities with the human in terms of gastrointestinal anatomy, metabolism and physiology (Court et al., 2004). When compared with other farm animals, pigs acquire early sexual maturity, sizeable litter size and have a quick reproduction time. They also breed year-round, which makes them highly suitable for biomedical research programs (Polejaeva et al., 2016). Due to these characteristics and the anatomical and physiological similarities, and also their size (young pigs have a size and body weight similar to human adults), pigs are widely used as models in organ transplantation and other surgical procedures (Kahn et al., 1988; Chari et al., 1994; Martin et al., 1999; He et al., 2013; Spetzler et al., 2015; Vogel et al., 2017), or as preclinical models in drug discovery (Swindle et al., 2012; Segatto et al., 2017), and numerous naturally occurring and generated genetic models of human disease (Swindle et al., 2012; Polejaeva et al., 2016). Hence, and similarly to the areas of medicine described above, the pig is gaining traction as the large animal model of choice for the study of tissue engineering and regenerative medicine products and applications, and of biomechanic studies. A good evidence of this is the steep rise in the number of publications in these broad areas in the past 30 years (Cone et al., 2017).

### Drug Discovery and Toxicology

Traditionally, animal models used for preclinical testing of new drugs and toxicology studies have been rodents, mainly mice and rats, for the primary screening studies. Nonetheless, because translation from rodents into humans is often not fully realized, regulatory agencies also demand the use of non-rodent models. Pigs are increasingly being used as an alternative to dogs or primates, the previous nonrodent species of choice (Swindle et al., 2012). However, due to growing pressure from the public, there has been a drive for new alternatives. The pig has been favored as a suitable alternative, since they have many anatomical and physiological features valuable for translational research and are already well accepted as one of the gold standard surgical models (Swindle et al., 2012). In particular, the cardiovascular system, skin and digestive tract closely mimic the human. Due to these similarities the metabolism and toxic effects of chemicals and drugs in pigs may more closely resemble the effects in man than some other laboratory animals. The minipig has been introduced recently as another alternative (Dalgaard, 2015) which is frequently used due to its smaller size and easier handling for drug discovery and toxicology applications (Mcanulty et al., 2011), boosted by the publication of the RETHINK project (Forster et al., 2010). Furthermore, the porcine CYP450 system has been studied and partially described, and their metabolic pathways have been found to be relatively analogous to humans, with substantial overlap in substrate specificity (Skaanild, 2006; Murayama et al., 2009).

### Generated Genetic Models

With the advent of DNA recombination and gene editing technologies, modifying the pigs genome has enabled its use as a genetic model of numerous human diseases (Flisikowska et al., 2014; Yao et al., 2016). This is reflected in the multiple pig strains developed to study, amongst others, cancers, Duchenne muscular dystrophy, autosomal polycystic kidney disease, Huntington’s disease, spinal muscular atrophy, cystic fibrosis, hemophilia A, X-linked severe combined immunodeficiency, retinitis pigmentosa, Stargardt’s Disease, Alzheimer’s disease, various forms of diabetes mellitus and cardiovascular diseases (Flisikowska et al., 2014; Rogers, 2016; Yao et al., 2016; Perleberg et al., 2018). From these, the RAG2 or RAG2/IL2RG KO pigs are particularly relevant for biomedical research, since they can accept xenografts and/or human bioengineered tissue/organs (Boettcher et al., 2018).

### Transplantation Models

The pig has been used as a teaching and research animal model in surgery in the past decades. Starting in the 1990s, it became so prominent in academic and surgical training that it can be regarded as default model for non-survival surgical teaching classes, substituting the dog (Swindle, 2007). Its ubiquitous presence and use in academia, enabled also its widespread adoption in multiple models of liver, lung, heart, pancreas and kidney transplantation (Marubayashi et al., 1995; Martin et al., 1999; He et al., 2013; Fonouni et al., 2015; Mariscal et al., 2018). Furthermore, in transplantation medicine, the pig has also been proposed as xenograft donor, where porcine grafts have been transplanted into non-human primates with different degrees of success (Sachs et al., 2009; Griesemer et al., 2014). This has encouraged several research groups to target the porcine genome to eliminate the major xeno-antigen(s) recognized by human natural antibodies, in a so-called effort of humanizing the pig (Lai et al., 2002; Phelps et al., 2003; Petersen et al., 2011; Jeong et al., 2013). If ultimately realized, these procedures might enable the future xenotransplantation of porcine organs into humans as the main approach for transplantation medicine. Efforts are currently being taken to reduce the risk of viral zoonosis from porcine endogenous retrovirus (PERV), either by pharmacological treatment of PERV or by inactivating it with gene editing tools (Denner, 2017; Niu et al., 2017). Finally, other efforts have been concentrated on porcine uterus, urethra, kidney or liver bioengineering for transplantation (Baptista et al., 2011; Sullivan et al., 2012; Campo et al., 2017; Simoes et al., 2017). All these are an important testimony of the relevance of the pig as a vital translation research animal model.

### Skin

The minipig has been used as a model in the development of dermatological products (Mitra et al., 2015; Yamamoto et al., 2017), and more recently, as a model for microbiome studies (Ericsson, 2019). As omnivores with an analogous gastrointestinal tract to humans, the well-characterized fecal microbiota of young and adult domestic pigs and other strains used in research also offers compositional resemblances to that of humans (Pedersen et al., 2013b; Zhao et al., 2015). Remarkably, many of these strains are used to investigate diet-induced obesity in genetically susceptible individuals and the same modifications (e.g., an increase in the ratio of Firmicutes to Bacteroidetes) observed between lean and obese humans are emulated in these pig models during the development of obesity (Pedersen et al., 2013a).

### Musculoskeletal Disorders

In this particular area of biomedicine, the pig is experiencing a higher increase in adoption when compared to other large animal models (Cone et al., 2017) and several studies have been published assessing interspecies and interstrain differences in the anatomy and biomechanics of tissues and joints and their applicability in tissue engineering and regenerative medicine studies. Porcine models have a long history of use for studying the biomechanics of specific joints like the knee or the temporomandibular joint (TMJ), and specific tissues, including bone, cartilage, and ligaments (Xerogeanes et al., 1998; Sweigart et al., 2004; Proffen et al., 2012; Murphy et al., 2013; O’leary et al., 2017). Hence, the pig has been used with success to test the efficacy of bone substitute biomaterials (Li et al., 2015) and in osteochondral defect studies (Gotterbarm et al., 2008; Meng et al., 2020). Similarly, extensive research has been conducted with the pig in tendon and ligament repair as reviewed by others (Carpenter and Hankenson, 2004).

Pigs have also been used recently as a model of amyotrophic lateral sclerosis (ALS). This research has been based on the use of transgenic pigs with a mutated human copper/zinc superoxide dismutase 1 gene that mimics the human neurodegenerative disease in these pigs (Chieppa et al., 2014; Yang et al., 2014). Similarly, a pig model of Duchenne muscular dystrophy (DMD) has been created by Klymiuk et al. by deleting DMD exon 52 in male pig cells by gene targeting. The offspring generated by nuclear transfer exhibit absence of dystrophin in skeletal muscles, progressive dystrophic changes of skeletal muscles with impaired mobility, muscle weakness and a maximum life span of 3 months due to respiratory impairment (Klymiuk et al., 2013).

### Additional Considerations Regarding Pigs

Pigs suffer from porcine malignant hyperthermia also known as porcine stress syndrome which is characterized by hyperthermia triggered by stress, certain anesthetic agents or intense exercise and may lead to sudden death (Nelson, 1990). Some of the most important advantages and disadvantages of using pigs as model animals are summarized in Table 4.

**TABLE 4:** Advantages and disadvantages of the pig as a model.

**Advantages**	**Disadvantages**
Size of the pigs: Multiple and longitudinal measurements possible	Ethical concerns but minor compared to companion animals
Functional equivalence of various diseases in men and pigs	Special facilities needed for housing, surgery, imaging, necropsy
Genetic variation between breeds (cfr human population), moderate genetic variation within breeds (naturally occurring diseases)	Non-weight-bearing postoperatively is not feasible but can modulate with location
Imaging plus validated scoring approaches available (esp. for orthopedics)	Costs
Arthroscopy (also second look) plus validated scoring approaches	

## Companion Animals as Animal Models

### General Considerations

The importance of companion animals to serve as models for human disease has received significant attention through the One Health initiative which aims to “break through the species barrier” in a drive toward a better link between medical and veterinary research for the benefit of both the human and veterinary patient (Christopher, 2015).

While the definition of companion animals covers a range of animals this article extends only to the dog and cat as models, as they share remarkable similarities with the human and provide unique opportunities for developing advanced therapeutics.

One of the main reasons why dogs returned as a focus of genetic research is related to the specific population structure that has been created over the past 150−200 years.

To fully appreciate and exploit the biomedical potential of dogs (both as pets and as experimental animals), some insight into the unique canine population structure is necessary. Domesticated dogs were subjected to rigorous breeding selection, for instance for behavioral traits and/or specific morphological features such as excessive muscle formation, short limbs or a specific coat color (Larson et al., 2012). Illustrative for this process is the extreme size variation, by far the largest of all mammals known, ranging from less than 1 kg for Chihuahua dogs to over 70 kg for Irish wolfhounds and Neapolitan Mastiffs. This selection process was intensified in the last two centuries and resulted in isolated genetic populations of dog breeds (Parker et al., 2010). Whereas the genetic variation over the various breeds remained intact, the reduced genetic variability within breeds worked as a genetic amplifier and offers “genetic dissection microscope” for research (Lindblad-Toh et al., 2005; Parker et al., 2010; Larson et al., 2012; Van Steenbeek et al., 2016). Together with the selection for unique traits, an increased risk for the development of specific inheritable disorders arose within breeds, providing physiologically relevant models corresponding to human conditions. To make the best out of the current situation may be to exploit the downside of inbreeding as a gene-discovery instrument for causative and modifier genes involved in complex diseases and/or rare diseases.

### Canine Inherited Copper Toxicosis

The trace element copper is indispensable for critical biochemical processes such as enzyme function, for instance cytochrome c oxidase (part of the respiratory enzyme complex) or superoxide dismutase (conversion of superoxide radicals into molecular oxygen or hydrogen peroxide) (Inesi, 2017). Since copper is a transition element (reduced as Cu+ and oxidized as Cu^2^+) its Jekyll and Hyde character becomes evident in the involvement in chemical reactions leading to the production of reactive oxygen species. In a Fenton reaction, Cu^+^ catalyzes the formation of the highly reactive hydroxyl radical (OH**^.^**). In the converse Haber-Weiss reaction Cu^2+^ inactivates the damaging superoxide radical O_2_. Therefore, regulation of its intracellular free concentrations is of utmost importance and needs to be controlled within very narrow limits (Kim et al., 2008). Several inherited copper-related diseases are diagnosed in men such as Menke’s Disease (copper deficiency disorder), Wilson Disease (WD, copper accumulation), and the very rare Indian childhood cirrhosis (Tanner, 1998), endemic Tyrolean infantile cirrhosis (Muller et al., 1996), and idiopathic copper toxicosis (Scheinberg and Sternlieb, 1996). These all are rare diseases posing specific obstacles for researchers aiming to dissect molecular pathways and for rational drug design. These obstacles include limited financial resources compared to diseases affecting large numbers of patients, smaller patient cohorts for clinical phase 1−3 studies, difficulties for properly matched case-control studies in genetics and molecular signaling studies.

Copper disorders also affect sheep and dogs (Twedt et al., 1979; Haywood et al., 2001; Fuentealba and Aburto, 2003). Deleteriously increased levels of hepatic copper are described in a number of dog breeds including Bedlington terriers, Skye terriers, West-Highland White terriers, Doberman, Dalmatians and Labrador retrievers (Twedt et al., 1979; Haywood et al., 1988; Thornburg et al., 1996; Thornburg, 1998; Webb et al., 2002; Hoffmann et al., 2006). In 1999 genetic mapping studies revealed that the copper toxicosis locus within Bedlington terriers was located on canine chromosome 10. 3 years after positional cloning a 13kB deletion covering exon-2 of the murr1 gene was identified as the causative mutation for Bedlington terrier copper toxicosis (Van De Sluis et al., 1999, 2002). The causative role of murr1 mutations in WD is a matter of debate. Stuehler et al. found an association between murr1 mutations and WD, whereas two other papers did not detect a correlation between murr1 mutations and WD (Stuehler et al., 2004; Lovicu et al., 2006; Wu et al., 2006). This novel gene product, currently called COMMD1 (COpper Metabolism Murr1 Domain-containing protein 1) had no known function at the time it was discovered, and the mechanism of action related to hepatic copper accumulation remained enigmatic. The discovery that COMMD1 and ATP7B interact intracellularly revealed a mechanistic link between COMMD1 protein and copper toxicosis, later confirmed for the Menkes Disease protein ATP7A (De Bie et al., 2007; Vonk et al., 2012).

The discovery of the COMMD1 mutation and subsequent investigations into functions of COMMD1 is an intriguing example for a useful exploitation of inbred dog strains to reveal novel molecular and genetic pathways. Genetically speaking the big advantage of canine genetics to benefit human genetics is the ease to discover modifier genes. This is a needle-in-a-haystack technology in men even today, but the specific genetic population structure in inbred dogs clearly facilitates this approach.

Labrador retrievers are among the most popular breeds in the Western world.

It was already known for a long time that approximately one in every three first-line relatives of Labradors retrievers with copper toxicosis had elevated copper levels (Hoffmann et al., 2006). This pushed investigations into whether or not Labrador retrievers were new model animals for WD and as a consequence propelled genetic studies (Fieten et al., 2014). A SNP based genome-wide association study aiming to discover the genetic background of inherited copper toxicosis in Labrador retrievers included over 200 Labrador retrievers (154F, 81 M cases; 37F and 22 M as replication cohort) in the Netherlands that were genotyped on the 170k SNP Illumina Canine HD Bead Chip (Fieten et al., 2016). For details on the mechanism of action of these mutations the readers are referred elsewhere (Fieten et al., 2016). Approximately 12% of the phenotype can be explained by two mutations identified in Labrador retrievers. Since mutations in these genes were already described in copper-related disorders, it remains to be seen what other as-yet-unidentified genetic mutations will be discovered.

This genetic study clearly illustrates the power of the canine model. Explaining 12% of the phenotypic variation with an ample 250 dogs doesn’t even remotely resemble the number of human patients used to explain similar percentage for age at menarche, Inflammatory Bowel Disease (IBD) and Rheumatoid arthritis (RA) for which over 100,000 individuals were included (Elks et al., 2010; Okada et al., 2014; Liu et al., 2015).

The examples prove that due to the specific population structure of inbred dog breeds, genetic studies can be successfully performed even for rare and/or complex genetic diseases.

In order to investigate COMMD1-deficient dogs as a preclinical model for liver stem cell transplantations, a breeding colony of five COMMD1 deficient dogs was created on a Beagle background and followed for over 4 years (Favier et al., 2011, 2012; Favier et al., 2015). This model for inherited copper toxicosis has some practical features specifically relevant for pre-clinical studies that aim to investigate surgical procedures. In contrast to mouse models, that are sacrificed for every liver measurement, the dogs’ size allowed for a true longitudinal study permitting liver biopsy sampling twice a year.

### Heart Disease

The most prevalent non ischaemic cardiomyopathies in humans are hypertrophic cardiomyopathy (HCM) and dilated cardiomyopathy (DCM), reported to affect 1 in 500 and 35 in 100,000 people, respectively (2017, Heron, 2016). Arrhythmogenic ventricular cardiomyoapthy (AVC) is also recognized as an important and distinct form of cardiomyopathy. Together they are associated with mechanical and/or electrical dysfunction and manifestations of the disease can range from microscopic alterations in cardiomyocytes and cardic fibroblasts to heart failure (which results in inadequate tissue perfusion and fluid retention) and arrhythmia which may cause sudden death. In veterinary species HCM is the most common feline cardiac disease affecting around 1 in 15 cats and DCM is the second most common cardiac disease in dogs and can affect a wide variety of breeds including the Doberman where its cumulative prevalance is as high as 44%. AVC has been comprehensively described in the Boxer breed at the molecular, cellular and clinical levels. All three cardiomyopathies share striking pathological and clinical similarities with the human disease. While there has been progress in the management of the symptoms associated with these cardiomyopathies in human patients, the actual disease processes remain a challenge to treat as there are few therapies that target the underlying pathology. There has therefore been an emphasis on the use of regenerative cellular therapies, although most studies have focused on ischaemic myocardial disease using mesenchymal stem cells (MSCs) derived mostly from bone marrow or adipose tissue. Stem cells derived from myocardial tissue have more recently been developed and have been tested in a number of induced disease models. A comparison of MSCs and cardiosphere derived cells (CDCs) suggests that CDCs are more efficacious in their ability to regenerate the myocardium (Li et al., 2012) and phase 1 clinical trials using autologous CDCs show encouraging results (Bolli et al., 2011; Makkar et al., 2012; Malliaras et al., 2014).

The development of cell-based approaches in the feline and canine clinic will have significant benefits for translation in human cardiomyopathy treatment.

### Human and Feline Hypertrophic Cardiomyopathy

Hypertrophic cardiomyopathy is the most common cardiomyopathy in both humans and cats with a prevalence of approximately 0.1−0.2% and 16%, respectively (Maron et al., 1995; Payne et al., 2010; Semsarian et al., 2015; Husser et al., 2018). There is increasing literature that supports the cat as an animal model of human HCM and evidence suggests it is essentially the same disease in both species (Maron and Fox, 2015). HCM is characterized by left ventricular hypertrophy in the absence of systemic causes and can result in heart failure and/or sudden death. In humans genetic mutations are identified in 60% of HCM cases, mainly in genes encoding sarcomeric proteins (Cahill et al., 2013). HCM in the cat is also considered to have a familial cause although only two causative mutations have so far been identified (Maron and Fox, 2015), in contrast several hundred have been identified in human patients. Both of the feline mutations occur in the cardiac myosin binding protein C (MYBPC3) gene, one of which occurs in the Maine Coon breed (A31P mutation) and the other in the Ragdoll breed (R820W mutation) (Meurs et al., 2005, 2007). It is of interest to note that one specific non-truncating mutation, MYBPC3/R820W, that occurs in Ragdolls has been identified in a human family with HCM (Ripoll Vera et al., 2010; Borgeat et al., 2014). The role sarcomeric mutations play in the development of HCM in non-pedigree cats requires further investigation.

The underlying molecular pathogenesis driving HCM remains to be elucidated although a common pathway is thought to exist in both humans and cats in which altered calcium handling within the myofilaments enhances calcium sensitivity, causing maximal force production and energy deficiency promoting mitochondrial dysfunction, cell death, fibrosis and cardiomyocyte hypertrophy (Huke and Knollmann, 2010; Marston, 2011; Song et al., 2013; Robinson et al., 2018).

Studies using myocardial tissue from a cat homozygous for the MYBPC3/R820W mutation suggest that increased myofilament calcium sensitivity can occur in the absence of haploinsufficiency, which is common feature in human MYBPC3 mutations (Messer et al., 2017). Increased myofilament calcium sensitivity was also seen in other HCM affected cats of unknown genotype but not in unaffected cats. An additional feature of the study was that the calcium sensitivity of the sarcomere is uncoupled from the phosphorylation status of troponin I, although it remains unclear how mutations outside the troponin complex cause this uncoupling phenomenon. The reasons clearly are complex but the similarities at the molecular level show the cat to be a highly relevant natural disease model for human HCM for deciphering the mechanisms. Targeting the disease with Epigallocatechin-3-gallate, for example can reverse troponin I phosphorylation uncoupling in cat HCM (Messer et al., 2017) which has been replicated in human HCM samples (Sheehan et al., 2018).

Such studies highlight the need to identify detailed molecular mechanisms for precise drug targeting. However, there are practical limitations with obtaining sufficient heart tissue and the survival of isolated primary cardiomyocytes is poor. Induced pluripotent stem cells (iPSC) or embryonic stem cells (ESCs) represent an alternative and robust source for preparing cardiomyocytes. The development and use of human ESCs represents an ethical dilemma and while less of an issue in veterinary species, there are only two reports of ES-like cells from cats, but these do not replicate indefinitely in culture unlike true ES cells. iPSCs on the other hand do not have the concerns associated with ESCs and can be relatively readily prepared from somatic cells.

Feline iPSCs have recently been reported for the first time by our group, the development of which represents a significant step in the generation of iPSC derived cardiomyocytes from a veterinary species (Dutton et al., 2019). It paves the way for generating further cell lines from feline patients carrying the HCM causing MYBPC3/R820W mutation to test novel therapeutics for modifying the disease. iPSCs can further be manipulated with technologies such as Clustered Regularly Interspaced Short Palindromic Repeats (CRISPR) to enable targeted genetic manipulation of both normal and diseased patient cell lines (Cai et al., 2018; Sasaki-Honda et al., 2018).

iPSCs derived from patients with HCM or iPSCs with a genetic mutation inserted using CRISPR to model HCM, display characteristics of hypertrophic cardiomyocytes in culture (Mosqueira et al., 2018) suggesting the suitability of the approach in establishing cell models of HCM. The availability of feline iPSC lines will enable dissecting out the molecular mechanisms of HCM enabling targeted drug screening where promising molecules can be rapidly assessed in the feline clinic with the potential of swift translation to human patients.

### Human and Canine Dilated and Arrhythmogenic Ventricular Cardiomyopathy

DCM is the third most common inherited myocardial disease in humans with an estimated prevalance of 0.35% and some 2.5 million cases globally affected^6^. It is the second most common cardiac disease in dogs and accounts for 10% of canine cardiac diagnosis (Egenvall et al., 2006). As with feline HCM there are remarkable similarities in the pathophysiology of DCM between human and dog. Although it is a heterogenous disease it is characterized by progressive enlargement of the left ventricle that leads to reduced systolic function, congestive heart failure and a variety of arrhythmias. Underlying causes include systemic disorders such as hypertension and atherosclerosis in humans but is also now recognized as a primary genetic disorder that may manifest with or without accompanying predisposing factors. Giant dog breeds such as the Great Dane and Newfoundlands are at risk and a genetic basis has been proposed in some dog breeds including the Doberman Pinscher and Boxer in which the disease is both common and severe with a cumulative prevalence in European Dobermans >8 years of age of 44% (Mausberg et al., 2011; Simpson et al., 2015a,b). A genetic deletion in the Pyruvate Dehydrogenase Kinase 4 (PDK4) gene has been reported. PDK4 is critical in regulating mitochondrial energy metabolism as the genetic deletion predisposes affected individuals to developing DCM as it results in chronic energy attenuation (Meurs et al., 2012). More recently a missense variant in the titin gene has been reported in affected Doberman pinscher dogs negative for the PDK4 mutation. The Boxer breed has a distinct form of cardiomyopathy that closely resembles AVC in humans (Vischer et al., 2017). A causative mutation in the striatin gene has been identified in Boxer dogs in the United States but this was not seen in the UK population (Meurs et al., 2010; Cattanach et al., 2015). The role of genetics in other dog breeds with DCM remain to be better described.

Histopathological observations of the myocardium show that canine cardiomyopathy displays either an attenuated wavy fiber type and fibro-fatty infiltration type (Tidholm and Jonsson, 2005) with the latter highly similar to AVC in humans. These findings emphasize the comparable pathological changes and clinical presentation between the two species (Basso et al., 2004; Meurs et al., 2014; Vila et al., 2017). The pathophysiologic mechanism underlying AVC is thought to involve mechanical and electrical decoupling and cardiomyocyte apoptosis (Wess et al., 2010) which with the fibro-fatty replacement of the myocardium are considered primary drivers for risk of arrhythmia and sudden cardiac death. Dogs that survive develop progressive ventricular dilation and systolic dysfunction leading to congestive heart failure (Wess et al., 2010; Meurs et al., 2014).

There have been efforts to use stem cells for the treatment of cardiac disease in humans spurred by observations that the adult heart processes regenerative ability (Condorelli et al., 2001; Nadal-Ginard et al., 2003). A number of clinical trials are under way or completed using adipose or bone marrow derived mesenchymal stem cells (MSC) although these are predominantly for ischaemic disease. One published study in Doberman pinchers with DCM administered allogeneic adipose derived MSCs that were virally transfected to overexpress stromal derived factor-1 to enhance homing and engrafting capabilities of endogenous MSCs to the myocardium (Pogue et al., 2013). Although no significant improvements in survival rates were found at 2-year follow up, the study demonstrated that the dog model of naturally occurring DCM can be utilized to overcome a number of challenges for regenerative therapies. There is increasing interest in CDCs as they appear to possess a superior ability to regenerate the myocardium (Li et al., 2012) compared to MSCs. CDCs are a heterogenous cardiac stem cell population which display features typical of stem cells such as forming clones, self-renewal and commitment to multiple lineages (Johnston et al., 2009; Chimenti et al., 2010; Cheng et al., 2014; Hensley et al., 2015). The use of CDCs clearly is not practical because of the need to sample from the patient and also because of expansion of cells from a diseased individual which adds to patient risk, time and treatment costs. Allogeneic cells offer an alternative off-the-shelf-product but risks include immunological complications that may lead to graft versus host disease. Work in a rodent model and other induced disease models suggests allogeneic CDCs are non-immunogenic (Malliaras et al., 2012). Allogeneic CDCs have been tested in a small clinical trial in dogs affected with DCM (Hensley et al., 2017) and no significant adverse effects were reported. Nevertheless, the process of cryofreezing of cell stocks may potentially alter intrinsic properties of the cells as has been shown for MSCs (Moezzi et al., 2005). Effects such as chromosome abnormalities resulting in abberrant cellular activity and risk of tumorigenesis may compromise their clinical use. However we have demonstrated that cryopreservation of dog CDCs does not alter their immunophenotype and cellular characteristics (Dutton et al., 2018a). Furthermore, we have shown at a molecular level that canine CDCs are also immune- privileged similar to the immunomodulatory function of MSCs (Dutton et al., 2018b) and cryopreservation retains this property suggesting they are safe to use *in vivo*.

### Musculoskeletal Disorders in Companion Animals

#### Osteoarthritis

Dog models have long been used to study joint disorders particularly osteoarthritis. The canine model for osteoarthritis has been more commonly used than the horse, sheep or goat model (Mccoy, 2015). One of the reasons might be the easier post-operative management and follow up using various exercise regimes on e.g., treadmills (Mccoy, 2015). While there are some similarities in cartilage anatomy between humans and dogs, the standing angle in the hindlimb in dogs is much larger. This should be considered when biomechanical aspects are compared and evaluated (Mccoy, 2015). As stated previously the cartilage thickness in dogs is 0.6−1.3 mm and cartilage defects are considered to have a critical size at a minimum diameter of 4mm. Experimental OA is preferably induced in the stifle joint (Pond and Nuki, 1973; Marijnissen et al., 2002; Kuroki et al., 2011), whereas naturally occurring disease is also common in the elbow or hip joint with an estimation prevalence of OA affecting 20% of adult dogs (Mccoy, 2015).

With respect to osteoarthritis dogs are divided in two classes, non-chondordystrophic (NCD) and chondrodystrophic (CD) dogs. The last group presents with disproportionally short limbs, caused by aberrant endochondral ossification of long bones. Dachshunds are typical examples. The molecular mechanisms of this short limb phenotype is associated with a retrogene insertion of the FGF4 fibroblast growth factor 4 gene. This leads to elevated levels of FGF3 signaling. Interestingly, whereas CD dogs are more prone to intravertebral disc degeneration (IVDD), the insertion of the retrogene renders short-limb dogs less likely to develop OA in comparison with NC-dog (Tellegen et al., 2019). These examples emphasize the need to carefully select for a specific dog breed for musculoskeletal investigations.

#### Intervertebral Disc Degeneration

Despite walking on four legs in contrast to men walking on two only, both species develop intervertebral disc degeneration with great similarities and similar prevalence. Link-N is a protein involved in proteoglycan stabilization (beneficial) and is highly homologous between men and dogs. However, neither human link-N nor canine link-N can protect cultured canine intervertebral disc cells form degeneration, whereas human link-N improved glycosaminoglycn deposition in human and bovine chondrocyte-like cell cultures (Bach et al., 2017).

In a classcial pre-clinical study a controlled release system for the COX-2 inhibitor celecoxib (cyclooxygenase-2) was tested in a dog model for IVDD (Tellegen et al., 2018). Since celecoxib prevented IVDD progression and reduced inflammation, follow-up studes will be conducted in a clincal study aiming to eliviate the chronic pain associated with low back pain.

#### Cranial Cruciate Disease and Meniscal Injury

Naturally occurring cranial cruciate disease has been studied extensively in veterinary medicine (Cook, 2010; Bergh et al., 2014). It can therefore be stated, that the pathophysiology differs between injuries in humans and canines, because dogs typically suffer from degenerative ruptures (Comerford et al., 2011) as compared to acute traumatic injuries seen in humans. To study new treatment approaches and validate their success, experimental models with artificially severed cruciate ligaments should be employed (Bozynski et al., 2016).

Dogs also suffer from naturally occurring meniscal pathologies and hence lend themselves as potential translational models to study mechanisms of degeneration or for testing new treatment strategies (Krupkova et al., 2018). The canine meniscus has comparable anatomic features (vascularization, cellularity, collagen structure) and similar permeability to the human (Sweigart et al., 2004*;* Deponti et al., 2015). However, some differences between canine and human menisci especially with regard to biomechanical properties such as the aggregate- and shear-modulus should be pointed out (Sweigart et al., 2004; Gupte et al., 2007).

### Nervous System

Cats often serve as models to study spinal cord healing and comparative aspects in neurosurgery (Barbeau and Rossignol, 1987*;* Bélanger et al., 1996*;* Abelew et al., 2000*;* Bouyer and Rossignol, 2003). Biomechanical motion analyses using treadmills and force plates as well as electromyography (EMG) are performed to evaluate spine kinematics and muscular properties following experimentally induced spinal cord or cerebral lesions.

### Additional Considerations Regarding Companion Animals

Dogs and cats are companion animals and pets and as such subject of unprecedented love and care in our society. Therefore, studies involving dogs and/or cats raise more ethical debate than other animal studies. However, most studies in these animals use clinical cases seen in veterinary hospitals and clinics, which highlights the importance of this underused resource for research. Some of the most important advantages and disadvantages of using dogs as model animals are summarized in Table 5.

**TABLE 5:** (a) Advantages and disadvantages of canine research in general and hepatology in particular. (b) Advantages and disadvantages of companion animals as models.

**Advantages**	**Disadvantages**
Size of the dogs: Multiple and longitudinal measurements possible	Ethical concerns – companion animals − More of a concern if experimental use
Functional equivalence of various diseases in men and dogs/cats	No canine hepatitis virus causally correlated with canine hepatitis
Large genetic variation between breeds (cfr human population), limited genetic variation within breeds (genetic magnifier glass)	Specific drug intolerances for specific breeds
Imaging plus validated scoring approaches available (esp. for orthopedics)	
Dogs: Arthroscopy (also second look) plus validated scoring approaches	Costs
Dogs: Objective weight-bearing of legs possible (force plate analysis)	Special facilities needed for housing, surgery, imaging, necropsy
Dogs: Size variations cover new-born human-size until adult size	Size variation, so drug dosing needs special attention
Clinical need, large patient population (pets) available	

## Conclusion

Companion animal and large animal models offer realistic naturally occuring disease models that more accurately evaluate safety and efficacy of new treatments as they share the heterogeniety of the human population including genetic and physiological variations and the complex interactions of these with the environment.

There are an increasing number of studies emerging from companion animals and large animal species that demonstrate they have much to offer to the human clinic in the quest for the next generation of drug or cell-based therapies and tissue engineering. The use of large animal models will enable greater attention to key questions. These include route of administration as it is not clear as yet which route(s) allow optimal engraftment of injected cells for different diseases. It also needs to be determined whether multiple injections will be more beneficial and if so the question arises whether there is an associated increase in risk of an adverse immune reaction. Cell therapies likely function via a paracrine mechanism and as such alternative approaches such as cell-free extracellular vesicle fractions or soluble factors, need to be explored that may reduce some risks posed by cell administration particularly of allogeneic cells.

For tissue engineered constructs implantation studies using animals with similar size and weight as human patients are crucial to test the implants under relevant biomechanical conditions.

To answer these questions pre-clinical trials with patient cohorts of sufficient size are required which need to be designed robustly to measure appropriate safety and efficacy readouts. Equivalent diseases in animals makes them not only relevant models which offer a more accurate evaluation of safety and efficacy of new treatments, but at the same time are potential beneficiaries of new treatment approaches. Hence, human and veterinary medicine can mutually benefit if one appreciates the similarities.

## Author Contributions

IR contributed to conceptualization, writing the manuscript, merging the parts contributed by other authors, and revision and editing of the manuscript. PB, AL-C, LM, MP, ES-F, LD, DC, and FS wrote the manuscript. FJ revised and edited the manuscript. JD wrote, revised, and edited the manuscript. LP conceived the idea and wrote, revised, and edited the manuscript. All authors contributed to the article and approved the submitted version.

## Conflict of Interest

The authors declare that the research was conducted in the absence of any commercial or financial relationships that could be construed as a potential conflict of interest.

## Footnotes

1 http://www.isscr.org/docs/guidelines/isscrglclinicaltrans.pdf

2 https://www.fda.gov/vaccines-blood-biologics/biologics-guidances/cellular-gene-therapy-guidances

3 https://ec.europa.eu/health/sites/health/files/scientific_committees/scheer/docs/scheer_o_004.pdf

4 https://omia.org/home/

5 https://goldcopd.org/wp-content/uploads/2016/04/GOLD-2018-WMS.pdf

6 https://www.bhf.org.uk/what-we-do/our-research/heart-statistics/heart-statistics-publications/cardiovascular-disease-statistics-2017
